# Mechanism of Electroacupuncture Regulating IRS-1 Phosphorylation in Skeletal Muscle to Improve Insulin Sensitivity

**DOI:** 10.1155/2021/8631475

**Published:** 2021-03-22

**Authors:** Shanshan Song, Rui Li, Bingyan Cao, Jingyi Zhang, Youngcho Kim, Bonggyu Kim, Xue Yu

**Affiliations:** ^1^Beijing University of Chinese Medicine, Beijing 100029, China; ^2^Beijing Xiyuan Hospital, Beijing 100091, China

## Abstract

**Objective:**

To explore the possible mechanism of electroacupuncture to improve insulin sensitivity in type 2 diabetes rats.

**Methods:**

Fourteen Zucker Diabetic Fatty (ZDF) rats were randomly divided into two groups: a model group and an electroacupuncture group, with 7 rats in each group. Seven Zucker Lean (ZL) rats served as a control group. All rats were fed with Purina #5008 for 4 weeks, and the electroacupuncture group received 4-week electroacupuncture intervention, while the control group and model group received no intervention. We measured fasting blood glucose (FBG) on the fourth weekend. After 4 weeks of intervention, the expression levels of insulin receptor substrate-1 (IRS-1) tyrosine phosphorylation, IRS-1 serine/threonine phosphorylation, and GLUT4 in quadriceps femoris muscles were detected by western Blot.

**Results:**

Compared with the model group, the electroacupuncture group had a lower level of fasting blood glucose, serum insulin level, and insulin resistance index (*P* < 0.05). The electroacupuncture group had lower IRS-1 serine/threonine phosphorylation than the model group, with the difference showing statistical significance (*P* < 0.05). Furthermore, the mean score (MS) of the control group showed the lowest phosphorylation expression, followed by the electroacupuncture group, while the model group had the highest level of phosphorylated protein expression. The level of IRS-1 tyrosine phosphorylation at Tyr895 sites was compared, and the result showed that there was no significant difference between the electroacupuncture group and the control group (*P* > 0.05), and the electroacupuncture group had higher phosphorylation expression than the model group (*P* < 0.05). Compared with the control group and the model group, the expression level of GLUT4 protein in the electroacupuncture group was significantly increased (*P* < 0.05).

**Conclusion:**

Electroacupuncture has the effect to improve the insulin sensitivity of type 2 diabetic ZDF rats by reducing fasting blood glucose, insulin level, and insulin resistance index, effectively up regulating the expression of GLUT4 protein in quadriceps femoris muscle. The mechanism is related to the regulation of skeletal muscle IRS-1 serine/threonine and tyrosine phosphorylation levels.

## 1. Background

Diabetes is a metabolic disease of the endocrine system caused by a combination of genetic and environmental factors [1]. Type 2 diabetes accounts for more than 90% of the affected population. With economic development, improved living standards, and changes in lifestyle, the prevalence of diabetes among obese people has increased significantly. Insulin resistance, an important mechanism for the onset of type 2 diabetes, is manifested by the decreased sensitivity of peripheral tissues and organs to insulin [2]. Skeletal muscle is the most important peripheral target organ of insulin. It is an important tissue for insulin-mediated uptake and utilization of glucose, where 80% of glucose circulating in the blood is taken up and metabolized by skeletal muscle. Therefore, it is particularly critical to study the mechanism of insulin resistance in skeletal muscle. Furthermore, the current hypoglycemic drugs all have certain side effects and adverse reactions, whereas acupuncture has been proven by a large number of studies to effectively interfere with type 2 diabetes. Therefore, exploring the mechanism of electroacupuncture to lower blood sugar and improve skeletal muscle insulin sensitivity is of great significance for the treatment of type 2 diabetes.

Under physiological conditions, elevated blood glucose can cause islet B cells to secrete insulin. The insulin receptor (InR) is activated on the skeletal muscle cell membrane after insulin is transported to the skeletal muscle. IRS-1 is the main component of skeletal muscle [3] and possesses tyrosine and serine kinase activity, where tyrosine is phosphorylated to open a series of downstream pathways so that GLUT4 is moved to the cell membrane to perform the biological activity of active transportation of glucose [4]. In insulin resistance, feedback stimulation leads to excess serine kinase activity, competitively inhibiting phosphorylation of tyrosine kinase, impeding normal phosphorylation of IRS-1, and reducing migration of glucose transporter GLUT4 to the cell membrane, resulting in glucose displacement into cells [5].

In this study, we investigated the effect of electroacupuncture on the phosphorylation level of IRS-1 in ZDF rats and observed whether electroacupuncture can promote the uptake of skeletal muscle glucose by regulating the phosphorylation of skeletal muscle IRS-1, which provides evidence for the mechanism of electro-acupuncture treatment in type 2 diabetes.

## 2. Experimental Materials

### 2.1. Experimental Consumables

A Roche ACCU-CHEK blood glucose meter (LOT: 10129770) and Roche blood glucose test strips (LOT: 24685431) were purchased from Roche (Basel, Switzerland). Acupuncture needles (LOT: 212161123) were purchased from Beijing Zhongyan Taihe Medicine Co., Ltd. (Beijing, China). An EA device (Yingdi KWD-808) was purchased from Changzhou Yingdi Electronic Medical Device Co., Ltd. (Hangzhou, China). Phospho-IRS-1 (Tyr 895) Antibody (LOT: #3070) and Phospho-IRS-1 (Ser 307) Antibody (LOT: #2381) were purchased from Cell Signaling Technology, Inc. (Boston, USA). GLUT4 Polyclonal antibody (LOT: 66846-1-Ig) and GAPDH Rabbit Polyclonal antibody (LOT: 10494-1-AP) were purchased from Proteintech Group, Inc. (Chicago, USA).

### 2.2. Experimental Animals

The experimental animals were 7 SPF-level male ZL (fa/+) rats with body masses of 140–160 g (2 months old) and 14 SPF-level male ZDF (fa/fa) rats with body masses of 140–160 g of the same age. All animals (license number SCXK (Beijing) 2016–0006) were purchased from Beijing Weitong Lihua Experimental Animal Technology Co., Ltd. Animals were housed in an animal facility at Beijing Xiyuan Hospital with a controlled environment of 23°C with air circulation, relative humidity of 60%, and 12 hours of day and night cycle lighting. The experiment started after 1 week of adaptive feeding. After adaption, all animals were fed with Purina #5008 (composition: crude protein ≥23.0%, crude fat ≥6.5%, crude fiber ≤4.0%, coarse ash ≤10%, moisture ≤11.0%, phosphorus ≥0.65%, and calcium∼0.75–1.25%; raw material composition: corn flour, shelled soy flour, wheat flour, fish meal, wheat bran, brewer's yeast, cane molasses, malt, beet powder, oat flour, soy flour, dehydrated alfalfa meal, minerals, and vitamins).

### 2.3. Model Preparation

After 1 week of adaptive feeding and 4 weeks of Purina #5008 feeding, with fasting blood glucose and OGTT 2 h blood glucose levels all above 11.1 mmol/L, the ZDF (fa/fa) rats became successfully prepared models. Altogether, 14 ZDF rats were included as model animals. The 14 ZDF rats were selected randomly according to blood glucose level by random number table method and were divided into two groups: model group and electro-acupuncture group, with 7 rats in each group. An additional 7ZL (fa/+) rats served as a control group.

## 3. Experimental Methods

### 3.1. Intervention

According to FBG measured after 4 weeks of Purina #5008 feeding, 7ZL rats and 14 ZDF model rats were randomly divided into 3 groups by random number table method. They were the control group (*n* = 7), model group (*n* = 7), and electroacupuncture group (*n* = 7). From the first day of grouping, all animals were continuously fed with Purina #5008.

#### 3.1.1. Control Group and Model Group

The animals were fixed in loose mouse bags and placed on the operating table for 25 minutes with no intervention.

#### 3.1.2. Electroacupuncture Group

The animals were fixed in loose mouse bags that permitted their limbs to be extended and held still for 5 minutes. Acupuncture needles of 0.13^*∗*^7 mm (Zhongyan Taihe brand of sterile acupuncture needles) were used to acupuncture the two sides of Zusanli (ST 36), Sanyinjiao (SP 6), Spleen Shu (BL 19), and Weiwanxiashu (EX B3). The needles were inserted to the depth of 4–6 mm and connected to the electroacupuncture instrument (one electrode was connected to the Weiwanxiashu (EX B3), while the other electrode was connected to the same side Zusanli (ST 36) to form a loop), which emitted a continuous wave frequency of 15 Hz and a current output intensity of 2 mA. It was suitable for the rat's muscle to be slightly stimulated without the rat squeaking. The needle was kept for 20 minutes, 6 times a week, for 4 weeks. All of the above interventions were performed daily between 13 : 00 and 18 : 00 for 4 weeks (Monday to Saturday). In the fourth week of the intervention, the rats were fasted at 20 : 00 on Saturday night, and then the FBG level of rats was measured at 8 : 00 on Sunday morning.

### 3.2. Sample Collection

After the intervention, all animals fasted at 22 : 00 in the evening. At 8 : 00 the next day, all animals were anesthetized with a 45 mg/kg dose of 2% pentobarbital sodium solution before being sacrificed by haemospasia. A 5 ml sample of blood was taken from the abdominal aorta and collected in a centrifuge tube and then solidified at room temperature. The serum was separated by centrifugation at 3000 r/min for 15 min at 4°C. The liquid supernatant was used to test FINS level with biochemical colorimetry. Quadriceps femoris muscle of all rats was collected on ice, frozen in liquid nitrogen, and stored in the refrigerator at −80°C for the western blot test.

### 3.3. Indicator Detection

The expression of IRS-1 phosphorylation and GLUT4 protein in skeletal muscle was detected by the western blot. For each group, 3 samples were selected randomly according to FBG taken from the −80°C refrigerator, with 100 mg of quadriceps femoris muscle per sample. Ripa lysate and protease inhibitor (phosphatase inhibitor was added to phosphorylation sample) were added to the sample, which was then homogenized on ice and left to stand for 30 minutes. After centrifugation at 4°C and 14000 rpm for 10 minutes, the supernatant was extracted. Then, the loading buffer was added and boiled for 10 minutes, cooled on ice and stored in a refrigerator at −20°C. The sample was electrophoresed on a SDS-PAGE gel (100 V for 75 min), then electrotransferred at 100 V for 75 minutes, and finally was diluted in 5% milk and was blocked at room temperature for 90 min. The primary antibody was incubated overnight. The membrane was stained the next day, and then after being incubated with the secondary antibody, the ECL (electrochemical luminescence) solution was used to expose the antibody reactions in the dark room. Fluor Chem software was used to analyze protein expression and phosphorylation levels. Using GAPDH as an internal reference, the protein expression level of GLUT4 and IRS-1 phosphorylation of the quadriceps femoris were calculated.

### 3.4. Statistical Analysis

Statistical analysis was performed using SAS 9.4 statistical software, and the results were expressed as mean ± standard deviation (‾*x* ± *s*). For comparison between multiple groups, if it conforms to the normal distribution and the variances are uniform, then the analysis of variance was used. If it does not conform to the normal distribution, the Wilcoxon rank sum test was used. The Pearson correlation test was used to analyze the statistical correlation between the two variables whose samples belong to the normal distribution. The difference was statistically significant with *P* < 0.05.

## 4. Experimental Results

**Figure 1:**
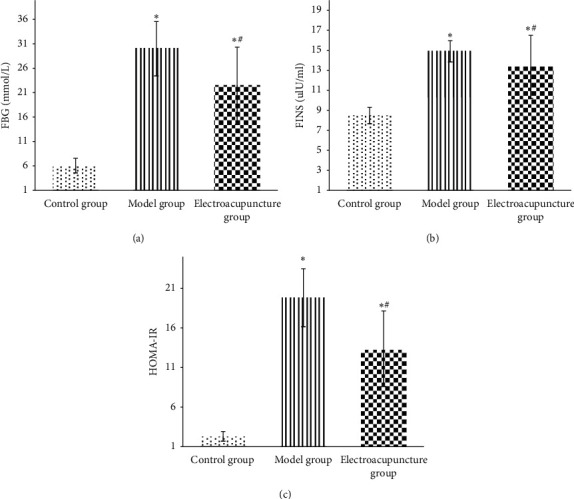
Changes in fasting blood glucose (a), insulin (b), and insulin resistance index (c) in each group (‾*x* ± *s*). Note: compared with the control group, ^*∗*^*P* < 0.05; compared with the model group, ^#^*P* < 0.05.

**Figure 2:**
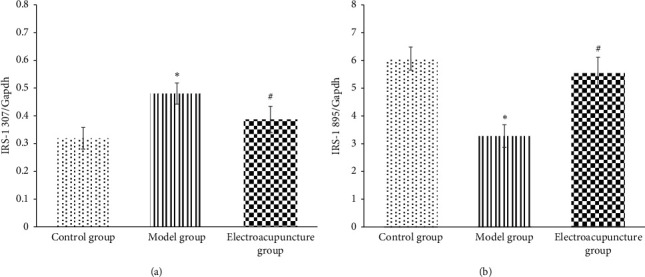
Changes in tyrosine (a) and serine/threonine (b) phosphorylation of IRS-1 in each group (‾*x* ± *s*). Note: compared with the control group, ^*∗*^*P* < 0.05; compared with the model group, ^#^*P* < 0.05.

**Figure 3:**
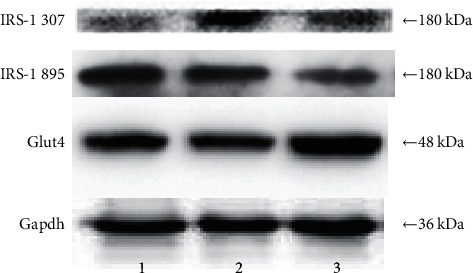
Comparison of expression levels of IRS-1 tyrosine, serine/threonine phosphorylated, and GLUT4 proteins in each group of rats. 1: control group; 2: model group; 3: electroacupuncture group; IRS-1 307: insulin receptor substrate-1 serine/threonine 307 phosphorylation site; IRS-1 895: insulin receptor substrate-tyrosine phosphorylation site.

**Figure 4:**
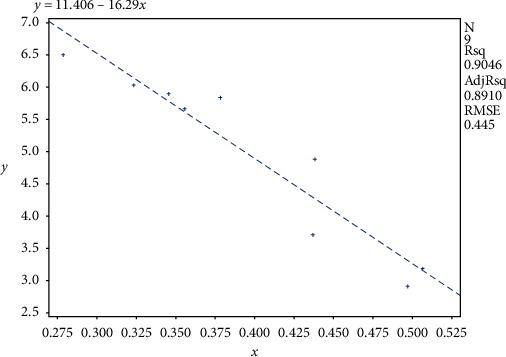
Pearson correlation analysis diagram of IRS-1 895 position and 307 position. *x*: IRS-1 serine/threonine 307 phosphorylation site; *y*: IRS-1 tyrosine 895 phosphorylation site.

**Figure 5:**
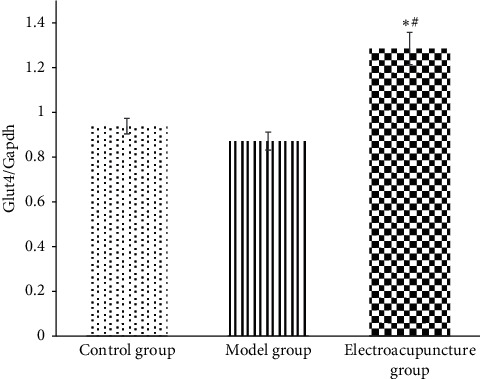
GLUT4 protein expression in skeletal muscle of rats in each group (*x* ± *s*; note: compared with the control group, ^*∗*^*P* < 0.05; compared with the model group, ^#^*P* < 0.05.

1. Results showed changes of fasting blood glucose (FBG), insulin (FINS), and insulin resistance index (HOMA-IR) in each group.  Figure 1 displays the fasting blood glucose level of the electroacupuncture group higher than that of the control group (*P* < 0.05); the fasting blood glucose of the electroacupuncture group is lower than that of the model group, and the difference is statistically significant (*P* < 0.05). The level of serum insulin in the electroacupuncture group is lower than that in the model group, and the difference is statistically significant (*P* < 0.05). Comparing the insulin resistance index, it can be seen that the HOMA-IR of the electroacupuncture group is lower than that of the model group, and the difference is statistically significant (*P* < 0.05). The insulin resistance index is consistent with blood glucose and insulin levels, thus indicating that electroacupuncture can alleviate skeletal muscle insulin resistance in type 2 diabetic rats.
2. IRS-1 tyrosine, serine/threonine phosphorylation levels in each group: it can be seen from Figures 2 and 3 that the phosphorylation level of IRS-1 serine/threonine in the model group is higher than that of the control group and the electroacupuncture group, and the difference is statistically significant (*P* < 0.05). There is no significant difference between the control group and the electroacupuncture group (*P* > 0.05). The mean score showed that the phosphorylation expression of the control group is the lowest, that of the electroacupuncture group is the second, and the model group had the highest expression. Comparing the levels of phosphorylation sites of three groups of IRS-1, tyrosine 895 showed that the phosphorylation expression of the model group is the lowest, and the difference is statistically significant compared with the control group and the electroacupuncture group (*P* < 0.05). There is no significant difference between the control group and the electroacupuncture group (*P* > 0.05). These results indicated that electroacupuncture could reduce the phosphorylation of IRS-1 serine/threonine in rat skeletal muscle and increase the expression of tyrosine phosphorylation in rat skeletal muscle. In addition, it can be seen from Figure 4 that Pearson correlation analysis shows that IRS-1 tyrosine and serine/threonine are negatively correlated (*r* = −0.951, *P* < 0.001)
3. GLUT4 protein expression in each group of rats: it can be seen from Figures 3 and 5 that compared with the control group and the model group, the expression level of GLUT4 protein in the electroacupuncture group is significantly increased, and the difference is statistically significant (*P* < 0.05). There is no significant difference between the control group and the model group (*P* > 0.05). The mean score showed that the electroacupuncture histone protein expression is the highest, followed by the control group, and the model group had the lowest expression. This data indicates that electroacupuncture has a certain positive effect on rat skeletal muscle glucose transport.

## 5. Discussion

The efficacy of acupuncture in the treatment of type 2 diabetes has been confirmed by a large number of clinical and animal experiments as well as recorded in Chinese ancient literature [6–8]. This experiment combined modern electroacupuncture treatment technology and the many years of clinical observation, to successfully study type 2 diabetic model rats. We follow the theory of correlations in neural networks to provide a basis for the selection of acupoints in this experiment [9]. It is believed that the back-shu points including Spleen Shu (BL 19) and Weiwanxiashu (EX B3) have the effect of regulating the corresponding visceral function by means of the close relationship between the deep spinal nerves and their organs [10, 11]. The Weiwanxiashu (EX B3) can effectively improve the pancreatic spleen function. In addition, Zusanli (ST 36) and Sanyinjiao (SP 6) are the most commonly used acupoints for the treatment of type 2 diabetes in clinical trial or animal experiment studies in China [12].

The results of this study show that the fasting blood glucose, serum insulin level, and insulin resistance index of the electroacupuncture group were higher than of the control group, with the difference being statistically significant (*P* < 0.05). Furthermore, the control group ZL rat genotype (fa/+) was different from the ZDF rat gene (fa/fa) in the model group and the electroacupuncture group, and the gene is considered to be the main cause of the large difference [13]. The electroacupuncture group had decreased results in the above three indicators compared with the model group (Figure 1). The control group indicated that electroacupuncture could reduce the blood sugar of type 2 diabetic rats and relieve the skeletal muscle insulin resistance. When peripheral blood glucose rises, insulin binds to cell membrane surface receptors, transports GLUT4 to the outer cell membrane, and binds to glucose to exert its biological activity [14]. From Figures 3 and 5, it can be seen that the expression level of GLUT4 protein in the electroacupuncture group was significantly increased (*P* < 0.05), indicating that electroacupuncture has the effect to reduce blood glucose and improve insulin sensitivity by upregulating the activity of GLUT4. As an endocrine hormone, insulin mediates metabolic regulation mainly through the activation of the phosphatidyl inositol 3-kinase (PI3K) pathway by an insulin receptor substrate protein [15]. IRS-1 phosphorylation directly affects PI3K pathway activity [16]. IRS-1 has two types of phosphorylation, including tyrosine and serine/threonine. In the case of insulin resistance, serine/threonine competitively inhibits tyrosine phosphorylation, of which serine/threonine 307 has an important negative regulatory effect on the insulin pathway [17]. IRS-1 tyrosine and serine/threonine were negatively correlated from the Pearson correlation analysis (Figure 4). Meanwhile, from Figures 2 and 3, it can be seen that the serine/threonine 307 phosphorylation site level of the electroacupuncture group was lower than that of the model group (*P* < 0.05), and the IRS-1 tyrosine 895 phosphorylation site level of the electroacupuncture group was shown to be significantly higher than the model group (*P* < 0.05). Electroacupuncture reduced the level of IRS-1 serine/threonine phosphorylation in T2DM rats' skeletal muscle and increased the expression of IRS-1 tyrosine phosphorylation in rat skeletal muscle. Thus, indicating that the alleviation of skeletal muscle insulin resistance is mainly through the regulation of the balance between IRS-1 phosphorylation, thereby reducing its impact on downstream pathways.

## 6. Conclusion

Electroacupuncture has significant effect of improving type 2 diabetic ZDF rats' insulin sensitivity, and effectively upregulating the expression of GLUT4 protein in skeletal muscle. The mechanism is related to the regulation of skeletal muscle IRS-1 serine/threonine and tyrosine phosphorylation levels.

## Data Availability

The data used to support the study are available in the supplementary files.

## Abbreviations

FBG: Fasting blood glucose
IR: Insulin resistance
InR: Insulin receptor
IRS-1: Insulin receptor substrate-1
MS: Mean score
T2DM: Type 2 diabetes
ZDF: Zucker diabetic fatty
ZL: Zucker lean.

## Supplementary Materials

Supplementary Materials Supplementary material includes the raw data of FBG, FINS, and HOMA-IR in “Sheet 1”, and the raw data of western blot in wb.
